# Overview of pathways and dynamic interactions of *c-*type cytochromes in *Geobacter sulfurreducens*

**DOI:** 10.1042/BST20260147

**Published:** 2026-09-18

**Authors:** Alexandre Almeida, Marta A. Silva, Jorge M.A. Antunes, Mafalda V. Fernandes, Leonor Morgado, Carlos A. Salgueiro

**Affiliations:** 1 Associate Laboratory i4HB – Institute for Health and Bioeconomy, NOVA School of Science and Technology, Universidade NOVA de Lisboa, 2829-516 Caparica, Portugal; 2 UCIBIO – Applied Molecular Biosciences Unit, Chemistry Department, NOVA School of Science and Technology, Universidade NOVA de Lisboa, 2829-516 Caparica, Portugal

**Keywords:** c-type cytochromes, Electrogenic bacteria, Extracellular electron transfer, NMR spectroscopy, protein-protein interactions, Redox partners

## Abstract

Extracellular electron transfer (EET) is a central process in the respiratory metabolism of electrogenic bacteria, enabling cells to exchange electrons with extracellular acceptors and donors. This capability has opened opportunities to exploit these bacteria in diverse practical applications, many of which would benefit from a precise understanding of the electron transfer (ET) events occurring within their respiratory chains. These chains rely on highly populated networks of *c*-type cytochromes that mediate electron flow across cellular compartments toward terminal electron acceptors. Although genomic, proteomic, biochemical, and structural studies have identified many ET components, defining their physiological redox partners and the directionality of electron flow remains challenging. This difficulty arises from the similar spectroscopic properties of key cytochromes, their transient interactions, and the complex overlap of their redox potential windows. In the present review, we examine how recently developed NMR-based strategies have been used to identify redox complexes established between cytochromes located in the inner membrane, periplasm, outer membrane, and extracellular conductive structures in the bacterium *Geobacter sulfurreducens*. These strategies exploited the distinct redox-dependent NMR fingerprints of individual heme groups, allowing direct detection of electron exchange, redox equilibria, and transient protein–protein interactions. Collectively, these studies reveal that ET pathways are not organized as single linear chains, but rather as dynamic and highly interconnected redox networks characterized by weak transient redox complexes and extensive promiscuity. This organization provides bacterial electron-buffering capacity, functional flexibility, and adaptability to changing environmental redox conditions.

## Introduction

Life is sustained by the regulated flow of electrons through networks of redox-active molecules. From cellular respiration to global biogeochemical cycles, electron transfer (ET) reactions drive conservation and matter transformations across all biological systems [1–4].In microorganisms, these processes are particularly diverse in dissimilatory metal-reducing bacteria, which have evolved specialized respiratory strategies that extend beyond conventional intracellular pathways [5]. Unlike most organisms, dissimilatory metal-reducing bacteria can use insoluble extracellular compounds as terminal electron acceptors without their uptake or assimilation [5,6]. Since these acceptors cannot cross the cell envelope, electrons must be transported from the cytoplasm across cell membranes to the extracellular environment [7,8]. This process, known as extracellular electron transfer (EET), expands the metabolic versatility of these bacteria and plays a central role in metal biogeochemical cycling. It has also considerable interest because of its potential in bioremediation, microbial fuel cells, and bioelectrosynthesis platforms [6,9–13]. Importantly, EET is reversible: electroactive microorganisms can either donate electrons to or accept electrons from extracellular compounds depending on environmental conditions. These applications emphasize the importance of understanding the molecular mechanisms underlying EET.

**Figure 1:**
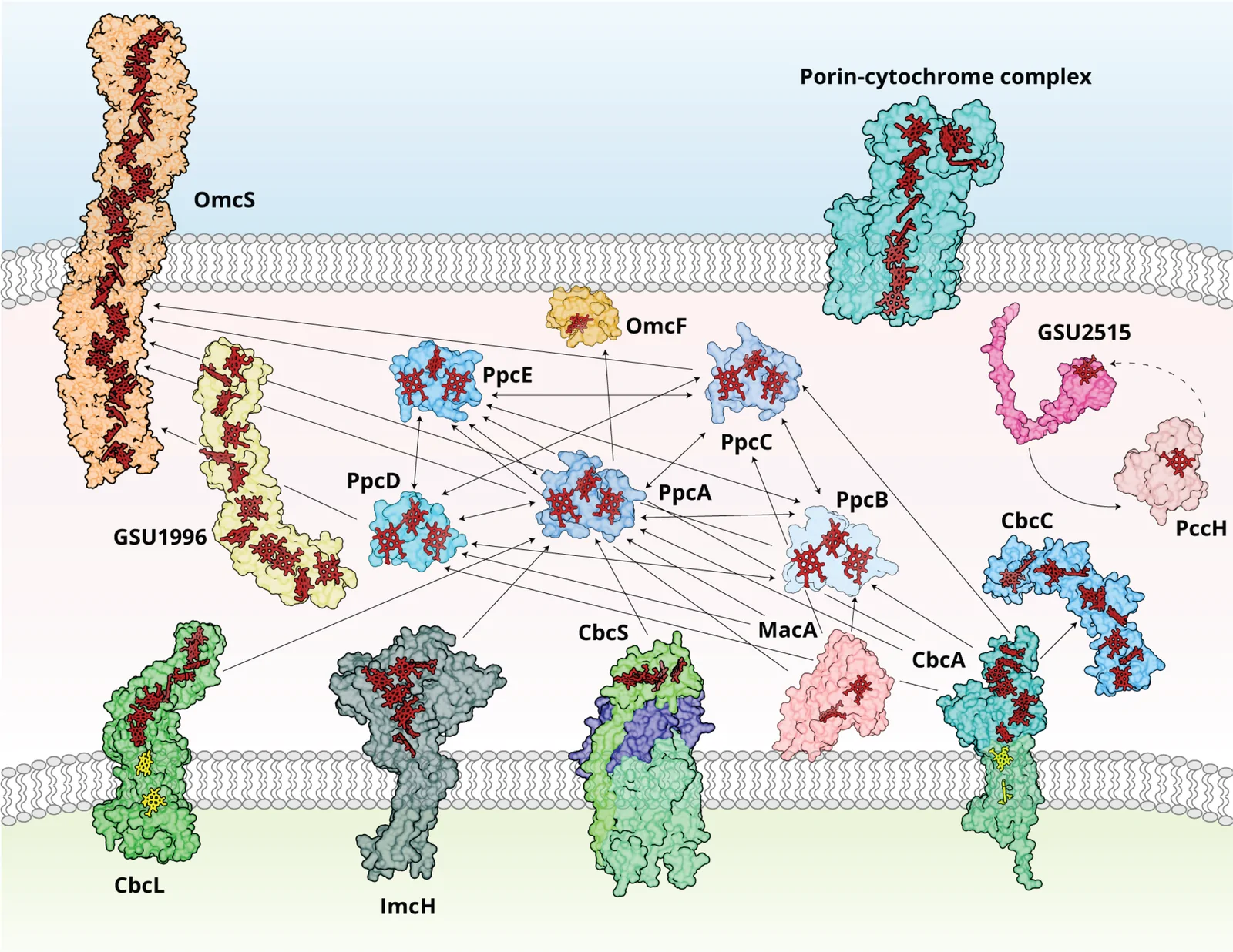
Redox interaction networks in *Geobacter sulfurreducens* Cytoplasm, periplasm, and extracellular space are distinguished by different background colors. Proteins lacking experimentally determined structures are represented by AlphaFold 3 models [33], whereas experimentally solved structures were used for PpcA (PDB 2LDO [34]), PpcB (PDB 3BXU [35]), PpcC (PDB 3H33 [36]), PpcD (PDB 3H4N [36]), PpcE (PDB 3H34 [36]), MacA (PDB 4AAN [37]), GSU1996 (PDB 3OV0 [38]), CbcA (PDB 2BPB [39]), OmcF (PDB 5MCS [40]), OmcS (PDB 6EF8 [41]), and PccH (PDB 4RLR [42]). The *b*- and *c*-type hemes are colored yellow and red, respectively. Solid arrows indicate observed ET, whereas the dashed arrow indicates no ET. All structures are represented as surface models and were rendered using ChimeraX [43].

Over the past decades, genomic, proteomic, and biochemical studies have significantly advanced the structural and functional characterization of the components involved in EET pathways [14–19]. Among dissimilatory metal-reducing bacteria, *Geobacter sulfurreducens* (*Gs*) has emerged as the principal model organism for investigating EET due to its metabolic versatility and the extensive repertoire of multiheme cytochromes encoded in its genome [20–24]. In *Gs*, cytochromes are strategically distributed across the inner membrane (IM), periplasm, and outer membrane (OM), forming an integrated network that transfers electrons from intracellular metabolism to extracellular acceptors (Figure 1). Such studies have established the current framework for EET, in which the identified redox components are organized into a functional model [21]. A summary of results from gene knockout and proteomic studies involving the cytochromes discussed in the present review is presented in Table 1. In this model, the electrons generated during the oxidation of organic substrates, primarily through the tricarboxylic acid cycle, are captured as NADH and transferred to the IM-bound menaquinone (MQ) pool, linking central metabolism to the respiratory chain [18,25,26]. This process is coupled to proton translocation across the IM, generating proton motive force and constituting a major energy-conserving step [18,27]. From the MQ pool, electrons are directed into distinct IM pathways according to the redox potential of the terminal acceptors. Three major pathways have been described: the ImcH-dependent pathway, active at relatively high redox potentials (above −100 mV relative to the standard hydrogen electrode, as reported throughout the present review) [28]; the CbcL-dependent pathway, operating at intermediate potentials (between −100 and −210 mV) [29]; and the CbcBA pathway (functioning below −210 mV) [30]. This redox-dependent branching enables *Gs* to maximize energy conservation by matching electron flow to the energetic landscape of available acceptors [31]. In addition, four other quinol-cytochrome *c* oxidoreductases—Cbc3/CbcVWX, Cbc4/CbcSTU, Cbc5/CbcEDCBA, and Cbc6/CbcMNOPQR—are predicted to facilitate ET from the MQ pool to the periplasm, suggesting additional complexity and possible redundancy. Their specific physiological roles remain unknown [30,32].

**Table 1:** Summary of data from gene knockout and proteomic studies on *Gs c*-type cytochromes

Predicted cellular localization	Protein	Proteomic studies	Gene knockout phenotype
Outer membrane	OmcS	Highest expression observed under metal oxide-respiring conditions (Fe(III) and Mn(IV) oxides) [32]	Growth in Fe(III) and Mn(IV) oxides affected [44]; No impact on current production [45]
	OmcF	More abundant during growth with Fe(III) oxides than Fe(III) citrate as electron acceptor [49]; No differential expression during Mn(IV) oxide respiration [32]	Fe(III) oxides [32], soluble Fe(III) [46], and U(VI) reduction [47] impaired; Current production [48] and *omcB* transcription decreased [46]
Periplasm	PpcA	The most abundant Ppc paralog during growth with fumarate, electrodes, and Fe(III) citrate [30]; Abundance decreases during late Fe(III) citrate reduction [30]; Detected during growth on Fe(III) oxides [49]; Up-regulated during Mn(IV) oxide respiration despite the absence of a corresponding knockout phenotype [32]	Single Δ*ppcX* mutants show no defect in Fe(III) citrate or Fe(III) oxide reduction and no increased sensitivity to H_2_O_2_; Quadruple *ppcA^+^, ppcB^+^ and ppcD*^+^ mutants supported WT metal reduction; *ppcC^+^ and ppcE^+^* exhibited impaired metal reduction, which is restored upon increasing their periplasmic abundance, demonstrating that all five Ppc paralogs are functionally interchangeable [50]; *ΔppcABCDE* showed markedly impaired, but not abolished, Fe(III) citrate and Fe(III) oxide reduction; Increased H_2_O_2_ sensitivity was observed in *ppcC^+^, ppcE^+^* and *ΔppcABCDE* mutants [50]; Strongly increases during late Fe(III) citrate reduction [30]; Selectively up-regulated during Mn(IV) oxide respiration [32]; Highly abundant during growth with fumarate and electrodes [30]
	PpcB	Detected during growth on Fe(III) citrate and Fe(III) oxides [30]; Abundance markedly increases during late Fe(III) citrate reduction [30,49]; Low abundance during growth with fumarate and electrodes [30];	
	PpcC	Low abundance during growth with fumarate, electrodes, and Fe(III) citrate [30]	
	PpcD	More abundant during growth on Fe(III) oxides than Fe(III) citrate [49]; Strongly increases during late Fe(III) citrate reduction [30]; Selectively upregulated during Mn(IV) oxide respiration [32]; Highly abundant during growth with fumarate and electrodes [30]	
	PpcE	Low abundance during growth with fumarate, electrodes, and Fe(III) citrate [30]	
	PccH	Up-regulated in current-consuming biofilms relative to current-producing and no-current conditions [51]	Inhibited ET from electrodes but did not affect ET to electrodes [51]
	GSU2515	Higher transcript abundance in current-consuming *versus* current-producing biofilms [51]; Highly abundant during growth with fumarate, electrodes and Fe(III) citrate; strongly enriched during late Fe(III) citrate reduction [30]	n.d.
	CbcC	Moderate expression during fumarate growth; Increased under electrode conditions; Strongly enriched in early Fe(III) citrate reduction, followed by decreasing markedly at late stage [30]	ET to insoluble Fe(III) impaired; Mn(IV) reduction unaffected [32]
	GSU1996	More abundant during growth with Fe(III) citrate *versus* Fe(III) oxide [49]	n.d.
	MacA	More abundant during growth with Fe(III) oxides *versus* Fe(III) citrate [49]; Up-regulated during Mn(IV) oxide respiration, despite the absence of a Mn(IV)-reduction phenotype [32]	Soluble and insoluble Fe(III) reduction impaired [32]; U(VI) reduction is affected [47]
Inner membrane	CbcL	Highly expressed during fumarate and electrode growth; Strongly down-regulated during Fe(III) citrate reduction, with near absence in late reduction stage [30]	No growth defect with fumarate and only a slight reduction in growth with soluble Fe(III) citrate [29]; Mn(VI) oxide reduction unaffected [29]; Reduction of low-redox-potential electron acceptors, including electrodes poised at −100 mV and Fe(III) oxide severely impaired, with strongly reduced rate and extent of Fe(III) reduction [29]; Fe(III) reduction ceases below −150 mV [29,52]; Associated with higher cell yield during Fe(III) reduction [32]
	ImcH	Enriched during electrode growth; relatively constant across fumarate and Fe(III) citrate conditions, with moderate increase in highly reduced Fe(III) citrate [30]	Reduction of electron acceptors with redox potentials > −100 mV abolished [28]
	CbcA	Up-regulated in Fe(III) oxide-grown cells [32]; Barely detected during growth with fumarate and early Fe(III) citrate reduction [30]; Strongly enriched during late Fe(III) citrate reduction and increased during electrode respiration [30]	No defect during growth with fumarate but fails to fully complete Fe(III) citrate reduction [30]; Fe(III) reduction ceases below −210 mV [30]; Reduction of insoluble Fe(III) (oxyhydr)oxide particles impaired, with stronger defects for lower-potential Fe(III) minerals [30]; Complementation requires both *cbcA* and c*bcB* [30]; Strains relying exclusively on CbcBA failed to reduce Fe(III) efficiently and were unable to grow on electrodes [30]
CbcS	Detected at low abundance during growth with fumarate, electrodes and Fe(III) citrate [30]; Abundance increases during late Fe(III) citrate reduction [30]; Up-regulated during growth on Fe(III) oxides compared with Fe(III) citrate [49] and down-regulated on Mn(IV) oxides [32]	Dissimilatory Fe(III) or Mn(IV) reduction not impaired [32]

Mutants lacking four Ppc homologs were named based on the single remaining gene in the native locus (e.g., Δ*ppcBCDE* = *ppcA*^+^, Δ*ppcABCD* = *ppcE*^+^). Early and late stages of Fe(III) citrate reduction correspond to 30% and 70% of Fe(III) reduced, respectively.

MacA is also an IM-associated diheme cytochrome *c* peroxidase implicated in oxidative stress response [53]. Gene knockout studies of MacA have reported impaired growth on both soluble and insoluble iron [32,53,54] and suggested a regulatory role in OmcB expression [54].

Regardless of the entry point, electrons are transferred to periplasmic redox proteins. The best characterized periplasmic ET proteins include the five triheme cytochromes from the PpcA-family (PpcABCDE), the dodecaheme cytochrome GSU1996 and the monoheme cytochromes PccH and GSU2515. These proteins generally act as mobile electron carriers mediating ET between the IM and OM, although some may also have specialized roles, including transient electron storage [55] or directional function within respiratory pathways.

Despite high sequence and structural similarity within the PpcA-family, thermodynamic studies have revealed distinct functional properties of the individual cytochromes [56]. For instance, PpcA and PpcD can couple electron and proton transfer, directly contributing to energy transduction, whereas this feature is absent in PpcB and PpcE [56]. Notably, the five cytochromes are constitutively expressed under diverse growth conditions [14,30,32,47,49,57], suggesting that multiple ET routes coexist, providing functional flexibility rather than simple redundancy. This is further supported by gene deletion studies showing that absence of all five cytochromes abolishes Fe(III) reduction, whereas strains retaining a single member partially or fully maintain this function [50]. Together, these findings highlight the essential and robust nature of periplasmic electron carriers, which ensures effective ET under varying environmental conditions [58].

At the OM, ET occurs over both short and long distances. Short-range transfer is mediated by large cytochromes organized into porin-cytochrome complexes, including Om(abc)B and Om(abc)C, ExtABCD, ExtEFG, and ExtHIJKL [59–62]. These complexes share a common architecture, typically comprising a periplasmic cytochrome, a transmembrane porin, and extracellular redox-active domain(s), enabling ET across the OM to extracellular compounds. Different complexes appear to be specialized for distinct extracellular electron acceptors, including metal oxides and electrode surfaces [31,32,59,63–65]. In addition to these complexes, lipid-anchored OM cytochromes, as OmcF, also contribute to EET [46]. This monoheme cytochrome is involved in Fe(III) reduction and current production [46,48] and has also been proposed to regulate the expression of other cytochromes [46].

In long-range ET, cytochromes OmcE, OmcS, and OmcZ assemble into polymeric filaments or nanowires that conduct electrons over micrometer-scale distances [41,66–68]. Their distinct heme packing arrangements support ET and functional specialization: OmcE and OmcS mediate the reduction of Fe(III) and Mn(IV) oxides [69,44], whereas OmcZ is essential for ET to electrodes [67,69,70].

Beyond electron donation, *Gs* can operate in a current-consuming mode by taking up electrons from extracellular sources and transferring them into the cell [71,72]. Although this process remains less well understood, it appears to involve a distinct set of redox components, reflecting the asymmetric organization of ET pathways [73]. Genomic and proteomic studies support this view, showing increased expression of the periplasmic cytochromes PccH and GSU2515 in current-consuming biofilms [73]. However, the molecular organization of the electron uptake pathway and its integration with the broader ET network remains largely unresolved.

Together, these findings show that ET in *Gs* is best viewed not as a linear sequence of reactions, but as a dynamic, interconnected network of redox proteins. The coexistence of multiple pathways, functional redundancy of key components, and the ability of cytochromes to engage in transient, promiscuous interactions indicate that ET is governed by a highly flexible and environment-dependent organization.

## Defining physiological electron transfer partners and pathways

Despite significant progress in identifying the proteins involved in ET and characterizing their structural and functional properties (Table 2), defining their physiological redox partners and the directionality of ET under different environmental conditions has proven challenging, limiting a comprehensive understanding of these pathways. These difficulties largely reflect the intrinsic properties of the low-spin *c*-type cytochromes that mediate ET. Their highly similar spectroscopic features, the relatively low production yields typically obtained for these cytochromes, which limit the amount of protein available for ET assays, the need to characterize both redox states, and the transient nature of their interactions hinder the application of conventional methods for identifying physiological redox partners (reviewed in [74]). Low-spin *c*-type cytochromes display nearly indistinguishable UV-visible spectra in both redox states, limiting the use of optical spectroscopy even when combined with stopped-flow kinetics measurements. On the other hand, each protein exhibits a characteristic NMR fingerprint arising from its heme group(s). Exploiting this property, NMR-based methodologies have recently been developed to directly monitor ET between *Gs* cytochromes [74]. This approach enables the identification of physiological redox partners *in vitro* and provides a straightforward strategy for dissecting complex respiratory chains, though the extent of the observed electron transfer reactions may be slightly affected *in vivo* by the relative abundance of the partners and the crowded nature of the shared cellular space. In the present review, we revisit studies that have applied this methodology to identify physiological redox partners in *Gs*, highlighting its broader potential for investigating complex respiratory chain networks.

**Table 2:** Electrochemical properties of cytochromes from *Gs*

Cellular localization	Cytochrome	*E*_app_ (mV)	Individual heme reduction potentials (mV)	Redox-active window (mV)	Reference
Outer membrane	OmcS	−130^b, c^	n.d.	−310 to +50	[75]
	OmcF	+127^c^	+127	+9 to +245	[76]
Periplasm	PpcA	−138	−154 (I); −138 (III); −125 (IV)^e^	−269 to −6	[56,35]
	PpcB	−143	−150 (I); −166 (III); −125 (IV)^e^	−279 to −16	[56,35]
	PpcC	−146	n.d.	−281 to −16	[77]
	PpcD	−148	−156 (I); −139 (III); −149 (IV)^e^	−278 to −7	[56]
	PpcE	−138	−167 (I); −175 (III); −116 (IV)^e^	−280 to +2	[56]
	PccH	−35	−35	−153 to +83	[78]
	GSU2515	−113^c^	−113	−231 to +5	[79]
	CbcC	−156	n.d.	−327 to +10	[39]
	GSU1996	−124^b^	-	−279 to +31	[22]
	MacA^a^	-	−241 (LP)^b^; (HP)^d^	n.d.	[37]
Inner membrane	CbcL	−194	n.d.	−366 to −36	[80]
	ImcH	−196^b, c^	n.d.	−431 to −38	[81]
	CbcA	−279	n.d.	−389 to −104	[39]
	CbcS	−151	n.d.	−302 to +19	[77]

Cytochromes are presented according to their predicted cellular localization. *E*app refers to the midpoint reduction potential, at which the oxidized and reduced fractions are equal. The values were obtained from redox potentiometric titration at pH 8 and 15°C, unless otherwise indicated. The redox-active windows were defined as the potential interval corresponding to 1-99% oxidation (or reduction). “n.d.” stands for not determined. The abbreviations LP and HP refer to low- and high-potential hemes.

a Data obtained from cyclic voltammetry experiments.

b Value determined at pH 7 (OmcS and GSU1996), 7.5 (MacA), and 7.6 (ImcH).

c Value determined at 25°C (OmcS, OmcF, PccH, and GSU2515) and 20°C (ImcH).

d Not experimentally determined. Comparison with homologous peroxidases suggests values ranging from approximately +320 to +450 mV.

e Heme numbering follows the convention established by the structural superposition with homologous tetraheme cytochromes *c_3_* [82].

## NMR-based monitoring of ET reactions

This methodology was designed to investigate the formation of a redox complex and the directionality of ET between PpcA and OmcF [74]. Both cytochromes contain low-spin *c*-type hemes with distinct and well-resolved NMR spectra in both redox states (Figure 3A). PpcA hemes are axially coordinated by two histidine residues (His–His), whereas OmcF heme exhibits His–Met coordination. In low-spin diamagnetic cytochromes, heme ring current effects alter the local magnetic field, shielding nuclei above the heme plane (axial ligands) and shifting their NMR signals upfield, while deshielding nuclei near the heme edges [83,84]. Consequently, ^1^H-NMR spectra are particularly useful for identifying heme axial ligands, especially methionine residues. Although methionine side chains typically resonate at 1–2 ppm, axial coordination to heme iron produces a characteristic high-field pattern, including a three-proton intensity peak near −3 ppm and up to four one-proton intensity peaks in the same region [84]. In the oxidized state, each heme contains an unpaired iron-electron causing greater signal dispersion and broadening. This paramagnetic contribution varies among cytochromes because it depends on the geometry of the axial ligands [85].

Figure 3A highlights the ^1^H-NMR fingerprints of PpcA and OmcF. In the reduced form, characteristic signals of a heme axial methionine are observed only for OmcF, including the εCH_3_ resonance at −2.83 ppm and four additional signals at −0.50, −1.23, −2.99, and −3.28 ppm. In the oxidized state, the two cytochromes exhibit different chemical shift dispersions, particularly in the low-field region (10–35 ppm). These distinct spectral signatures allow direct monitoring of ET between the proteins (Figure 3B).

The apparent midpoint reduction potential values (*E*_app_) of PpcA and OmcF (−138 mV [35] and +127 mV [76], respectively; Table 2) indicate that ET from PpcA to OmcF is thermodynamically favorable. However, this difference alone does not ensure ET, which also requires formation of a productive complex. To test this, oxidized OmcF (OmcF^ox^) was titrated into reduced PpcA (PpcA^red^) under anaerobic conditions at increasing protein ratios and, after incubation to reach equilibration, NMR spectra were recorded. At a 1:1 ratio, PpcA signals appeared in the 10–20 ppm region, while the εCH_3_ resonance of OmcF^red^ was detected at −2.83 ppm, demonstrating ET from PpcA to OmcF. However, PpcA was not fully oxidized, as PpcA^ox^ signals remained broadened, reflecting the presence of its three hemes. Progressive addition of OmcF led to gradual sharpening of these signals, with full oxidation achieved at a 1:3 ratio. To further confirm that the signal at −2.83 ppm corresponds to the axial methionine of OmcF^red^, the sample was exposed to atmospheric oxygen. In the resulting spectrum, both proteins became oxidized (bottom spectrum in Figure 3B) and the −2.83 ppm signal from OmcF^red^ disappeared. Considering these observations, this experiment provided direct evidence for ET between the two cytochromes and demonstrated the formation of a productive redox complex, with ET directionality consistent with the *E*_app_ values of the cytochromes.

## Identification of physiological redox partners in *Geobacter sulfurreducens*

The PpcA-OmcF system provided the first proof-of-principle for this NMR-based strategy to monitor interprotein ET. Building on this finding, the same methodology was applied to identify additional physiological redox partners in *Gs*, further expanding the characterization of the ET pathways.

### Periplasmic ET within the PpcA-family

The presence of structurally similar electron carriers within the same cellular compartment is particularly intriguing given the distinct respiratory pathways that coexist in *Gs*, where specific protein–protein interactions are expected to direct ET toward different terminal electron acceptors. The coexistence of five homologous triheme periplasmic cytochromes raises questions about how electron flow is organized among these proteins, and why the bacterium maintains this apparently redundant set of electron carriers. Members of the PpcA-family share similar amino acid sequences, structure and redox properties, and are co-expressed under multiple growth conditions [32,56,36,34]. Moreover, deletion mutant studies revealed extensive functional compensation among these cytochromes [50]. Together, these findings support the hypothesis that all PpcA-family members can interact with each other and function as a dynamic periplasmic ET network.

**Figure 2:**
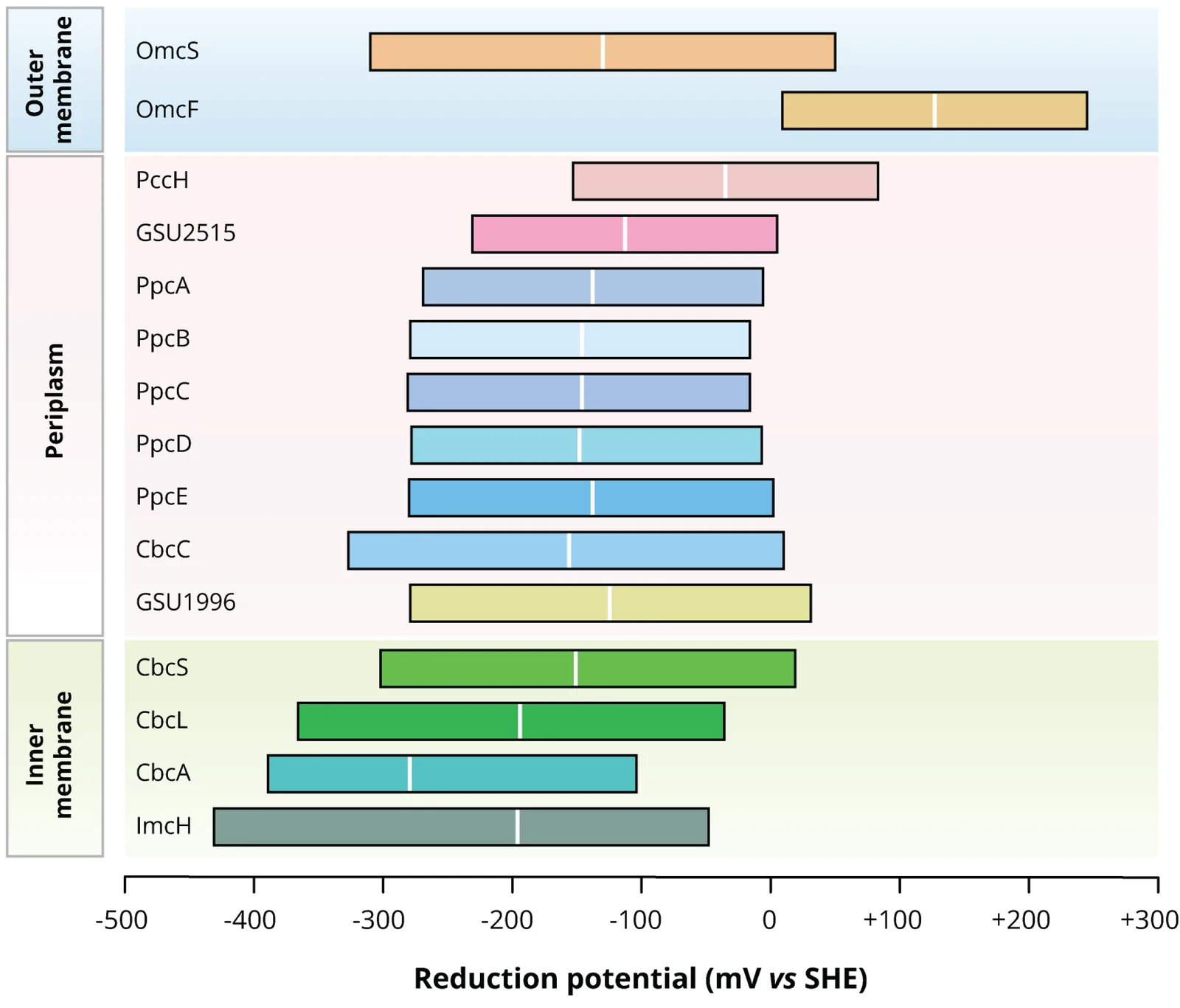
Redox-active windows of cytochromes involved in EET in *Geobacter sulfurreducens* Bars represent the experimentally determined reduction potential ranges of each protein, with bar length reflecting the width of the corresponding redox-active window (see also Table 2). Vertical white lines denote the apparent midpoint reduction potential (*E*_app_) of each cytochrome. For MacA, the redox-active window could not be experimentally defined and therefore is not represented.

NMR-monitored ET experiments showed that all pairwise combinations of the five cytochromes exchange electrons [58]. Mixing oxidized and reduced cytochromes at a 1:1 ratio under anaerobic conditions redistributed electrons between each pair, causing the characteristic resonances of both proteins to shift toward intermediate redox states and demonstrating interprotein ET (Figure 4A, left panel). These results are consistent with their similar reduction potential ranges and the substantial overlap of their functional redox windows (Figure 2 and Table 2), which facilitate reversible electron exchange. Together, these findings support a highly interconnected periplasmic redox network in which PpcA-family cytochromes dynamically redistribute electrons according to thermodynamic and physiological demands [58].

**Figure 3:**
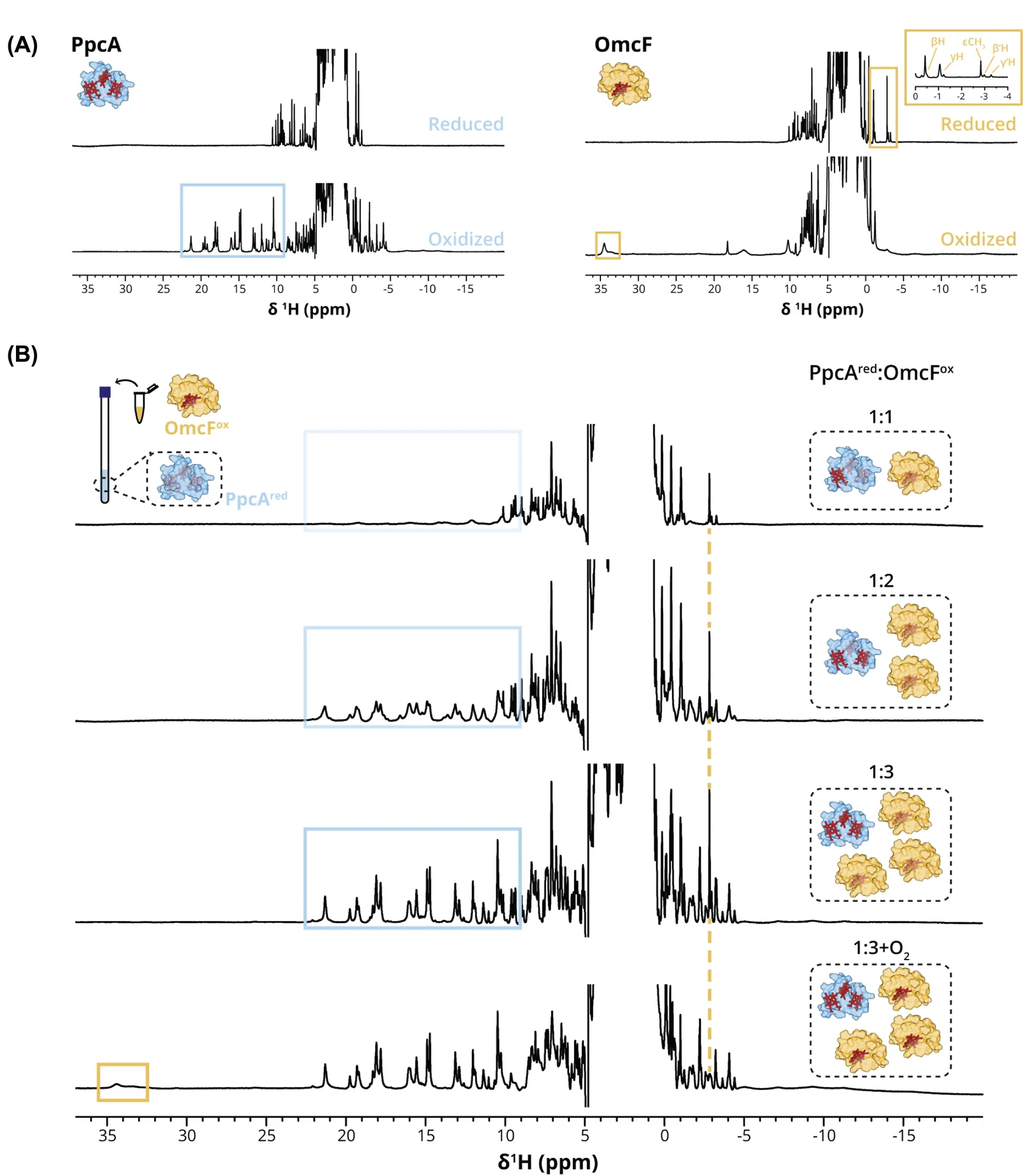
NMR monitoring of ET between PpcA and OmcF (**A**) 1D ^1^H-NMR spectra of reduced and oxidized PpcA (left) and OmcF (right). Blue (PpcA) and yellow (OmcF) boxes highlight the spectral regions containing the paramagnetically shifted heme methyl resonances in the oxidized state of each cytochrome. In the reduced spectrum of OmcF, the inset highlights the typical region of the axial methionine side chain signals and respective assignment. (**B**) 1D ^1^H-NMR spectra acquired following the addition of OmcF^ox^ to PpcA^red^ at increasing molar ratios (1:1, 1:2, and 1:3). The bottom spectrum was recorded after exposing the 1:3 mixture to molecular oxygen (1:3 + O_2_). The yellow dashed line indicates the signal of the εCH_3_ axial methionine from OmcF^red^. The decrease of the transparency of the blue boxes, together with the representative protein cartoons, illustrates the progressive oxidation of PpcA with the concomitant reduction of OmcF. In the representative protein cartoons, heme groups are colored red and pink to emphasize the oxidized and reduced states, respectively. Figure adapted from [74].

**Figure 4:**
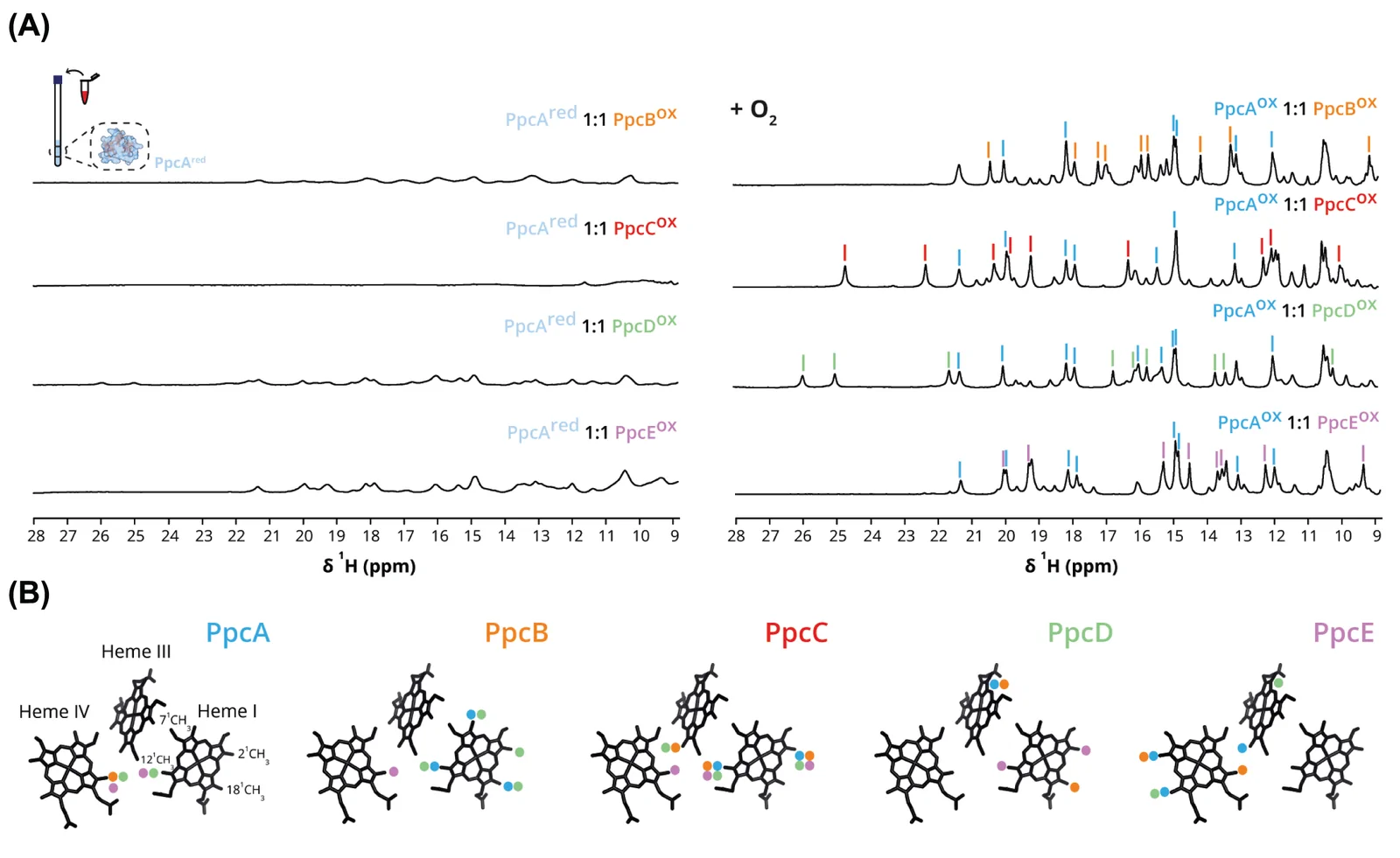
ET and interaction interfaces mapping within the PpcA-family (**A**) ET between PpcA and the remaining members of the family monitored by 1D ^1^H-NMR spectroscopy. Spectra acquired immediately after mixing PpcA^red^ with an equimolar amount of each oxidized partner (PpcX^ox^) are shown on the left, representing intermediate oxidation states of both cytochromes (see Figure 3A for the reference spectra of reduced and oxidized PpcA). The corresponding spectra recorded after subsequent exposure of each mixture to atmospheric O_2_ (PpcA^ox^:PpcX^ox^) are shown on the right. Each cytochrome is represented by a distinct color: PpcA (blue), PpcB (orange), PpcC (red), PpcD (green), and PpcE (pink). Colored bars identify the heme methyl resonances of each cytochrome. (**B**) Interacting interfaces of cytochromes PpcABCDE. The most affected methyl groups are indicated by a colored circle in the heme core of each cytochrome, using the same color code defined in panel A. For simplification, heme methyl groups are labeled only for heme I in the PpcA heme core, according to the IUPAC–IUB nomenclature for tetrapyrroles [86]. Figure adapted from [58].

The formation of ET complexes was further investigated by NMR chemical shift perturbation (CSP) experiments to map the interacting regions [87–89]. For the PpcA-family, CSPs were small, consistent with the weak affinities typically reported for transient ET complexes and in some cases involved opposite sides of the molecule [58]. Preferential perturbation of specific hemes was observed in some pairs—namely heme I in PpcB and PpcC, and heme IV in PpcE (Figure 4B). However, signals from all three hemes were affected in every pair, indicating that complex formation does not depend on highly specific interaction interfaces but rather occurs through multiple orientations and dynamic association modes.

Together, the ET and CSP results support a model in which PpcA-family members function as a dynamic, interconnected periplasmic redox network rather than as isolated components of linear ET pathways (Figure 1). The lack of highly specific interaction interfaces indicates that ET relies on weak and reversible association instead of stable complexes, enabling rapid electron exchange and high turnover between partners. Importantly, the cytochromes not only exchange electrons with one another but also remain functionally active at intermediate oxidation states, allowing each to act as either electron donor or acceptor according to the redox requirements. Combined with their similar structures and overlapping reduction potential windows, this intra-family promiscuity creates a dynamic pool of reducing equivalents that continuously redistributes electrons among multiple respiratory pathways. Thus, the apparent redundancy of the PpcA-family represents an adaptive strategy rather than an inefficiency, minimizing bottlenecks in electron flux and enabling responses to changing environmental conditions and terminal electron acceptors without extensive metabolic reprogramming [50,58].

### Periplasmic ET outside the PpcA-family

NMR CSP experiments were also used to probe interactions between PpcA and the dodecaheme cytochrome GSU1996 [90]. In the latter, the hemes are organized along a polypeptide chain in a nanowire-like arrangement, forming a modular structure with four domains (A, B, C, and D), each showing homology with PpcA-family cytochromes [38]. PpcA and GSU1996 form a transient redox complex at an interface that involves heme IV of domain C and heme I of domain D and is an interesting example of how cells can regulate electron traffic depending on their needs. When extracellular electron acceptors are available, PpcA can direct ET toward outside the cell. In their absence, GSU1996 may act as an intracellular electron reservoir, storing charge and supporting viability until suitable acceptors become available. Furthermore, the overlap between the redox potential values of GSU1996 and PpcA suggests a thermodynamically favorable bidirectional ET (Figure 2 and Table 2). Thus, electrons stored in GSU1996 could later be mobilized and discharged when terminal acceptors become available.

As discussed, *Gs* can also reverse the direction of electron flow, utilizing extracellular electron donors to support intracellular reduction processes. This versatility requires ET pathways that direct electrons from the cell exterior to the IM, a mechanism of interest since *Gs* is a promising platform for microbial electrosynthesis. Under current-consuming conditions, microarray analyses identified the genes for the periplasmic *c*-type cytochromes PccH and GSU2515 to be among the most highly expressed, suggesting key roles for these cytochromes in electron uptake. NMR-based experiments demonstrated a preferential electron flow from GSU2515 to PccH [79]. Indeed, when PccH^ox^ was added to GSU2515^red^, PccH became nearly fully reduced while GSU2515 was oxidized. In contrast, the reverse experiment caused only limited GSU2515 reduction, with PccH remaining largely reduced. This asymmetry agrees with the reduction potential profiles of the two cytochromes [79,78] (Table 2), confirming that ET from GSU2515 to PccH is thermodynamically favored. NMR CSP experiments further showed that GSU2515 and PccH form a weak-affinity, transient complex, with a binding interface near their heme groups [79]. Combined with the ET experiments, these findings support the existence of a physiological redox interaction between GSU2515 and PccH, linking extracellular electron uptake to periplasmic ET in current-consuming pathways.

Accumulating evidence supports a central role for the PpcA-family cytochromes in periplasmic electron trafficking. Their interactions with multiple redox partners suggest a broader function in connecting ET pathways across cellular compartments. In the following sections, we summarize the evidence for their interactions with cytochromes associated with the IM and OM.

## ET between PpcA-family and IM-associated cytochromes

### Quinol-cytochrome *c* oxidoreductases

Although the periplasmic ET network plays a central role in distributing electrons toward extracellular acceptors, the EET process begins at the IM. Given the localization of CbcL, ImcH, and Cbc3–6 complexes, these proteins are expected to functionally interact with periplasmic cytochromes. Identifying their periplasmic partners is therefore essential to understand how electrons are routed toward distinct respiratory pathways under different redox conditions.

**Figure 5:**
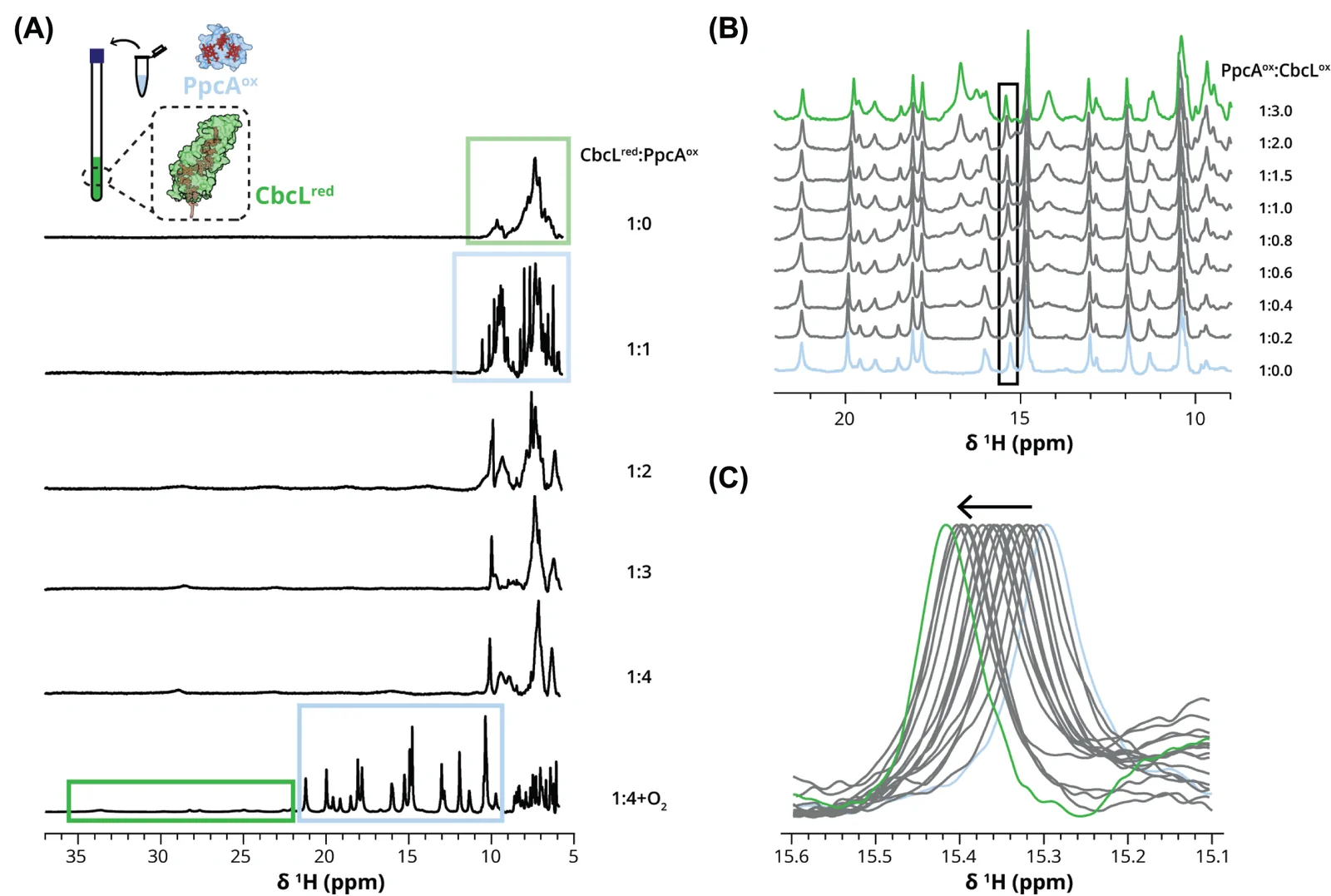
Functional interplay between CbcL and PpcA (**A**) ET between CbcL and PpcA monitored by 1D ^1^H-NMR spectroscopy during the stepwise addition of equimolar amounts of PpcA^ox^ to CbcL^red^ (top spectra), up to a 1:4 ratio. Green and blue rectangles highlight the characteristic spectral fingerprints of CbcL and PpcA, respectively. Translucent rectangles indicate the reduced-state fingerprints (CbcL, top spectrum; PpcA, second spectrum from the top), and opaque rectangles the oxidized-state fingerprints of both cytochromes. (**B**) NMR CSP of the PpcA’s heme methyl region upon the stepwise addition of increasing amounts of CbcL in the oxidized state. PpcA:CbcL molar ratios are indicated on the right of each spectrum. (**C**) Expansion of the resonance highlighted in panel B (black rectangle), corresponding to the methyl group 18^1^CH_3_ of PpcA heme I. Figure adapted from [80].

The periplasmic domain of CbcL contains 9 His−His coordinated *c*-type hemes and has a more negative *E*_app_ than the PpcA-family cytochromes (Table 2). From a thermodynamic perspective, these data support the hypothesis that the electrons are most likely transferred from CbcL toward periplasmic triheme cytochromes. The putative interaction was examined by NMR-monitored ET and CSP experiments with PpcA [80], the most abundant cytochrome in the periplasm of *Gs* [57]. The results demonstrated a thermodynamically favorable ET from CbcL toward PpcA (Figure 5A). Titration of CbcL^red^ with an equimolar amount of PpcA^ox^ caused significant changes in the ^1^H-NMR spectra, with signals characteristic of CbcL^ox^ becoming visible, whereas those of PpcA^ox^ were absent. Thus, electrons were transferred from CbcL^red^ to PpcA^ox^. Upon addition of further equivalents of PpcA^ox^, electrons became distributed between both cytochromes, populating intermediate oxidation states. Complete oxidation of CbcL was not achieved even at CbcL^red^:PpcA^ox^ ratios of 1:3 or 1:4, indicating that the redox equilibrium favors electron sharing between the two proteins. This behavior agrees with the overlap of their functional redox windows (Figure 2 and Table 2) and suggests that both cytochromes act as dynamic redox reservoirs, sustaining continuous periplasmic electron flux in response to changes in the redox state of upstream and downstream partners. NMR CSP experiments also showed the formation of a weak, transient complex between the two proteins. Analysis of the PpcA spectra in the presence of CbcL revealed perturbations in heme methyl signals, especially in heme I methyl group 18^1^CH_3_ (Figure 5B,C). Although this study focused primarily on PpcA, the extensive overlap in redox properties and the functional interactions among them suggest that other members of the PpcA-family may also serve as physiological CbcL partners.

The CbcL and PpcA study provided the first direct evidence for ET between an IM-associated cytochrome and a periplasmic carrier. Recent studies on CbcA and CbcC extended this view by examining how ET may be relayed within the same respiratory complex and toward multiple periplasmic acceptors [39]. CbcA is the periplasmic component of the CbcBA quinol-cytochrome *c* oxidoreductase complex and contains seven *c*-type heme groups anchored to the IM through a C-terminal transmembrane helix. CbcC is a periplasmic dodecaheme cytochrome encoded downstream in the same gene cluster [30]. In the proposed complex, the membrane-integral subunit CbcB is expected to receive electrons from the MQ pool and transfer them to CbcA, which then relays them to periplasmic redox partners. Based on localization and redox properties, CbcC and PpcA-family cytochromes are plausible downstream electron acceptors for CbcA (Figure 2 and Table 2). NMR-monitored ET experiments supported these hypotheses [39]. Titration of CbcA^red^ with CbcC^ox^ yielded broad resonances between 11 and 25 ppm after the first CbcC^ox^ addition at a 1:0.25 ratio, with no signals from fully reduced CbcC [39]. Further CbcC^ox^ additions did not fully oxidize CbcA or reduce CbcC, indicating a reversible redox equilibrium in which electrons are shared between both cytochromes.

A second series of experiments examined ET between CbcA and the PpcA-family cytochromes [39]. In the CbcA^red^:PpcA^ox^ titration, the first addition led to the appearance of PpcA^red^ resonances, confirming ET from CbcA^red^ to PpcA^ox^. However, further PpcA^ox^ additions did not fully oxidize CbcA, even at ratios theoretically sufficient to exhaust its reducing equivalents. Instead, broad resonances characteristic of intermediate oxidation states persisted, and both cytochromes remained partially oxidized even after the addition of an excess of molar equivalents of PpcA^ox^ (1:4 ratio). Similar results were obtained for PpcBCDE, showing that CbcA can exchange electrons with all family members. As observed for the CbcL–PpcA interaction, the persistence of intermediate oxidation states agrees with the partial overlap of their functional redox windows, supporting reversible redox equilibria and dynamic electron buffering within the periplasm (Figure 1 and Table 2). Together, these results support the physiological role of CbcA as a redox interface that sustains continuous electron flow by accepting electrons from the quinone pool through the CbcBA complex and transferring them to periplasmic partners such as CbcC and PpcA-family cytochromes, which are then re-oxidized by downstream components of the respiratory chain.

Characterization of CbcS further supports a highly interconnected membrane–periplasm ET network in *Gs* [77]. CbcS is an IM-anchored tetraheme *c*-type cytochrome associated with the Cbc4 complex and is proposed to receive electrons from the iron-sulfur protein CbcT and transfer them to a periplasmic redox partner. Like CbcL and CbcA, CbcS has a more negative reduction potential than the PpcA-family cytochromes (Table 2), although the smaller redox separation suggests less favorable ET than for other IM-associated oxidoreductases. ET assays between CbcS^red^ and PpcA^ox^ showed broad resonances at approximately 17 and 27 ppm after the first PpcA^ox^ addition [77]. These signals correspond to the 7^1^CH_3_ and 18^1^CH_3_ methyl groups of heme IV in the first oxidation stage of CbcS, indicating that this heme is the preferred electron exit site. Further PpcA^ox^ additions produced additional low-field resonances characteristic of intermediate oxidation states, but neither cytochrome became fully oxidized or reduced. Again, these observations reflect the overlap between the functional redox windows of CbcS and PpcA, which prevents complete ET and allows electrons to remain dynamically distributed between both partners [77].

Finally, the last example of ET between IM-associated oxidoreductases and PpcA-family members is provided by ImcH [81]. ImcH comprises a N-terminal membrane domain with three transmembrane helices and a soluble domain with seven *c*-type hemes. In ^1^H-NMR-monitored ET experiments, adding PpcA^ox^ to ImcH^red^ generated resonances characteristic of PpcA^red^, confirming ET between the proteins. As increasing amounts of PpcA^ox^ were added, these signals broadened, and additional low-field resonances from intermediate oxidation states appeared [81]. Even at an ImcH:PpcA ratio of 1:3, both proteins remained in intermediate oxidation states, indicating the establishment of a redox equilibrium, consistent with the partial overlap of their redox-active windows (Figure 2 and Table 2). NMR CSP experiments mapped the PpcA binding region to the vicinity of hemes I and IV [81].

Overall, the findings described in this section reveal a highly interconnected IM–periplasm interface where functional promiscuity, overlapping redox windows, and transient interactions establish reversible redox equilibria, providing *Gs* with dynamic electron buffering capacity to sustain a continuous respiratory flux.

### Cytochrome peroxidases

**Figure 6:**
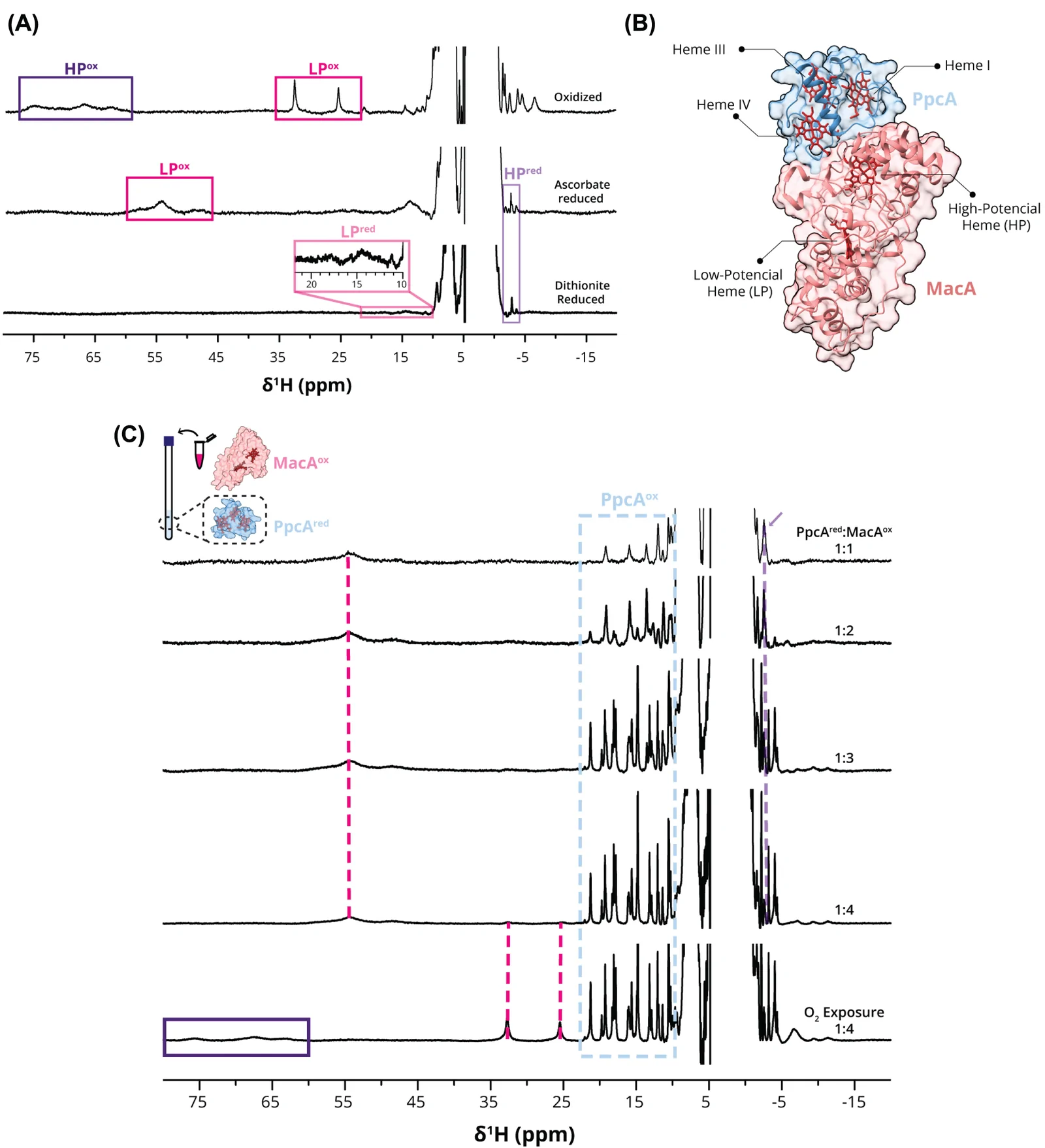
Characterization of the MacA–PpcA redox complex (**A**) 1D ^1^H-NMR spectra of MacA in three redox states (oxidized, ascorbate-, or dithionite-reduced). Characteristic signals of the high-potential (HP) and low-potential (LP) hemes are indicated. In the fully oxidized state, the His–Met coordinated HP heme undergoes rapid high-/low-spin exchange due to the weakly bound axial methionine, giving rise to broad resonances in the 60–80 ppm region, whereas the His–His coordinated LP heme remains low-spin, with resonances between 20 and 35 ppm. Upon ascorbate reduction, the HP heme is selectively reduced and adopts a low-spin state, as evidenced by the appearance of the axial methionine εCH_3_ resonance, while the LP heme becomes high-spin due to dissociation of the distal histidine, resulting in broadened resonances shifted to the 40–60 ppm region. Upon sodium dithionite reduction, both hemes are fully reduced: the HP heme maintains its low-spin state, whereas the LP heme maintains its high-spin state (see inset). Figure adapted from [92]. (**B**) Lowest-energy PpcA–MacA binding model calculated using the HADDOCK 2.2 webserver [93] based on PpcA backbone NH signals from NMR CSP data. The structures of PpcA (PDB 2MZ9 [94]) and MacA (PDB 4AAL [37]) are shown as ribbon representations in blue and pink, respectively, with heme groups depicted as sticks in red. The structures were rendered with ChimeraX [43]. Figure adapted from [95]. (**C**) 1D ^1^H-NMR spectra acquired at the different PpcA^red^:MacA^ox^ molar ratios. The NMR spectral features of both proteins are highlighted using the same colors as in panel A. The dashed blue rectangle highlights the PpcA heme methyl region monitored throughout the experiment (see also Figure 3A – left panel). Figure adapted from [92].

*Gs* was recently reclassified as aerotolerant based on genomic analyses identifying genes encoding proteins involved in aerobic respiration and oxidative stress protection, including superoxide dismutases and cytochrome *c* peroxidases [91]. The best characterized protein involved in periplasmic reactive oxygen species detoxification is the IM-associated diheme cytochrome *c* peroxidase MacA [37]. MacA adopts three redox stages: fully oxidized, partially reduced (ascorbate-reduced), and fully reduced (sodium dithionite-reduced) (Figure 6A). State-specific heme axial coordination generates distinct NMR fingerprints that were exploited in NMR-based ET experiments (Figure 6C). The results obtained showed that all PpcA-family cytochromes reduce the high-potential heme while leaving the low-potential heme oxidized, consistent with their redox potential values (high-potential in the range of +320 to +450 mV; low-potential, −241 mV; Table 2). NMR CSP studies (Figure 6B) mapped these transient interactions mainly to heme IV of the triheme cytochromes, except PpcC (heme I), and to MacA’s high-potential heme [92]. Thus, reduction of MacA’s high-potential heme by PpcA-family cytochromes supports the pivotal and ubiquitous role of this family in providing reducing power to a great array of redox partners in *Gs*, extending its function beyond EET to oxidative stress mitigation and enabling MacA to promptly eliminate harmful radical species [50].

## ET from PpcA-family proteins to OM and extracellular conductive cytochromes

Having crossed the IM and periplasmic ET network, electrons must ultimately traverse the OM to reach extracellular electron acceptors. Despite its central role in EET, this final stage remains poorly characterized, and biochemical evidence for direct ET between periplasmic and extracellular components is scarce. To date, besides the PpcA–OmcF interaction (Figure 3), ET has only been demonstrated between PpcA-family members and the OmcS nanowire [75].

**Figure 7:**
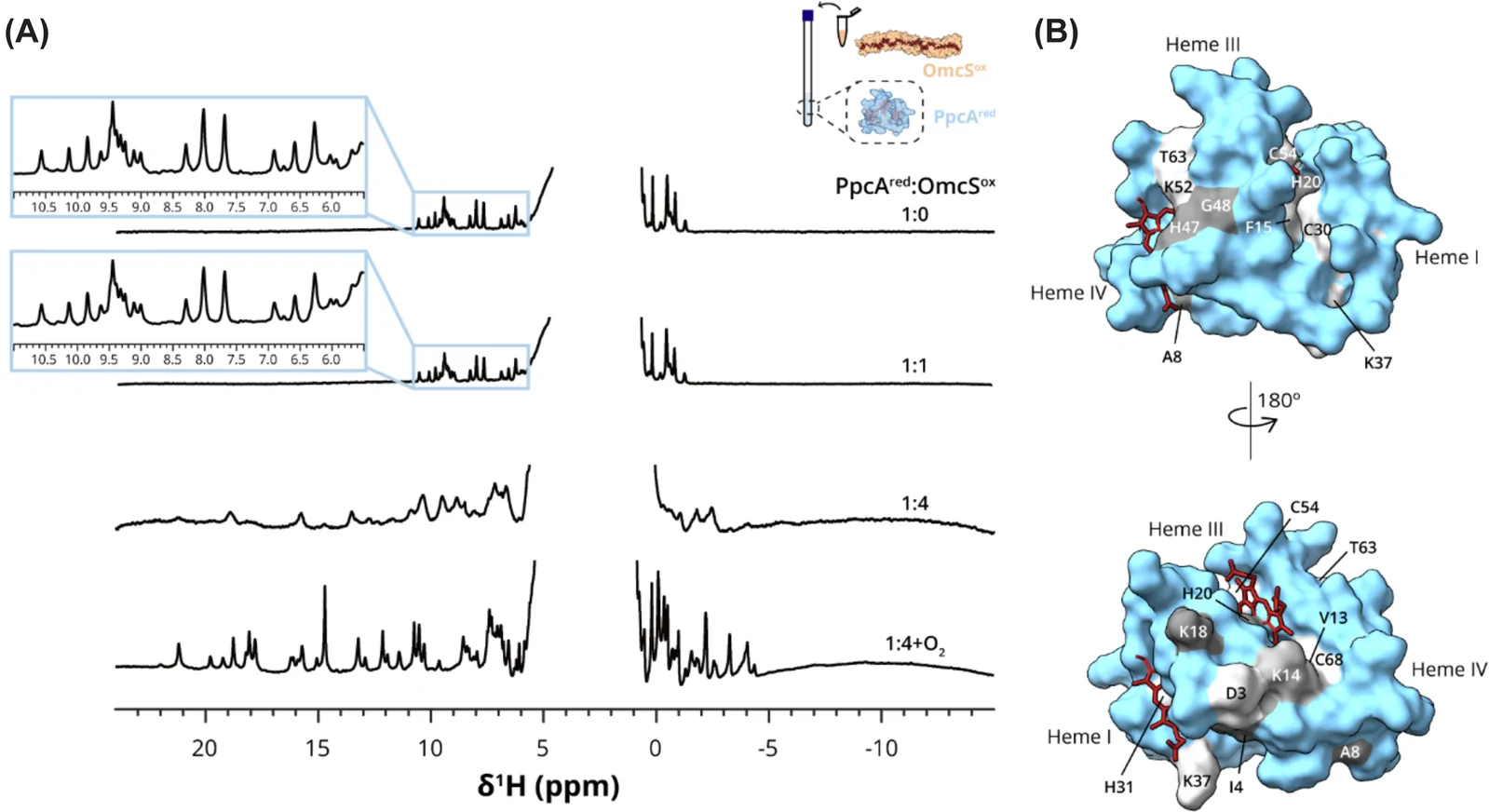
PpcA-mediated electron injection into OmcS nanowires (**A**) Oxidation of PpcA during ET to OmcS monitored by 1D ^1^H-NMR spectroscopy. Spectra obtained with increasing amounts of OmcS illustrate the progressive oxidation of PpcA, culminating in complete oxidation after exposure of the 1:4 mixture to molecular oxygen (bottom spectrum). For the sake of completeness, the spectrum of PpcA^red^ is included as a reference (top spectrum) for comparison with the 1:1 PpcA:OmcS mixture, confirming the absence of PpcA full oxidation. (**B**) PpcA (PDB code 2MZ9 [94]) residues interacting with OmcS. The most and least affected residues are highlighted in gray and light gray, respectively. Hemes are colored red. The structure was rendered using ChimeraX [43]. Figure adapted from [75].

The OmcS nanowire consists of polymeric monomers, each containing six His–His *c*-type hemes [41]. Its *E*_app_ closely matches those of the PpcA-family cytochromes (Table 2), consistent with their ability to exchange electrons with the OmcS nanowire, as shown by NMR-based ET experiments (Figure 7A). Interestingly, at a 1:1 PpcABCDE:OmcS heme ratio, a significant fraction of PpcABCDE remained reduced, indicating incomplete ET. Increasing OmcS to a four-fold excess relative to the reduced PpcABCDE cytochromes (4:1 heme ratio) generated NMR signals corresponding to more oxidized states of the PpcABCDE cytochromes, demonstrating a more extensive, although still incomplete, ET to the OmcS nanowire. The persistence of mixed redox states may provide a mechanism for maintaining a flexible distribution of electrons throughout the EET network, preventing complete reduction or oxidation of individual electron carriers. This behavior mirrors that observed for the IM-associated and periplasmic cytochromes, suggesting a common design principle that supports robust electron flux under fluctuating respiratory conditions.

NMR CSP experiments were also explored to identify the heme interface between PpcABCDE and the OmcS nanowire [75]. The most affected signals were from hemes III and IV (PpcA), heme I (PpcB), and hemes I and IV (PpcC, PpcD, and PpcE). These studies were consubstantiated by mapping the most affected residues in PpcA (Figure 7B). Overall, the results supported a transient, charge-complementary interaction in which PpcA uses multiple orientations for efficient electron injection into OmcS.

## Conclusions

EET in *Gs* emerges from the coordinated activity of a network comprising different redox-active proteins, rather than a single electron transport pathway. The studies presented in the present review show that electrons are transferred through multiple, partially overlapping routes involving IM-associated proteins, soluble periplasmic cytochromes, OM complexes, and extracellular conductive structures. This organization enables *Gs* to adapt electron flow according to the redox potential, availability, and localization of terminal electron acceptors or donors. A central feature of this network is the widespread involvement of multiheme *c*-type cytochromes. Their multiple redox centers, broad and overlapping reduction potential windows, and ability to populate intermediate oxidation states allow them to function as dynamic electron carriers, redox buffers, and connectors between distinct respiratory modules. The PpcA-family cytochromes exemplify this principle particularly well. Although they share high structural similarity and coexist in the periplasm, they do not appear to represent simple redundant components. Instead, their ability to exchange electrons with one another and with several redox partners suggests that they form a flexible periplasmic electron distribution network that minimizes bottlenecks, sustains continuous electron flux, and rapidly redirects reducing equivalents in response to changing physiological demands.

The NMR-based approaches reviewed here have also provided direct experimental evidence for several physiologically relevant ET partnerships (Table 3). They revealed electron exchange between different periplasmic cytochromes, within the PpcA-family, and between these cytochromes and IM-associated components CbcL, CbcA, CbcS, ImcH, and MacA, the latter involved in oxidative stress response. Finally, it was also shown that PpcA-family cytochromes can inject electrons into the conductive OmcS nanowire. These studies collectively support a model in which transient, weak-affinity, and often promiscuous protein–protein interactions underpin efficient ET. An important conclusion is that incomplete ET and the persistence of intermediate oxidation states should not be interpreted as experimental limitations. Rather, they likely reflect a biologically relevant property of the ET network. The partial overlap between the redox-active windows of many cytochromes enables reversible electron exchange and the establishment of redox equilibria, allowing electrons to remain dynamically distributed throughout the respiratory network. This provides buffering capacity and prevents the complete oxidation or reduction of individual components. Such a mechanism is well suited to an organism that frequently encounters fluctuating extracellular redox conditions.

**Table 3:** Redox partnerships tested within the EET network of *Gs*

Redox partners		ET	Preferential docking site	Reference
OmcF^ox^	PpcA^red^	+	n.d.	[74]
PpcABCDE^ox^	PpcABCDE^red^	+	Heme I (PpcB and PpcC) Heme IV (PpcE)	[58]
GSU1996^ox^	PpcA^ox^	n.d.	Heme IV (domain C) and Heme I (domain D)	[90]
PccH^ox^	GSU2515^red^	+	2^1^CH_3_ heme methyl (PccH)	[79]
GSU2515^ox^	PccH^red^	−	n.d	[79]
PpcA^ox^	CbcL^red^	+	Heme I (PpcA)	[80]
CbcC^ox^	CbcA^red^	+	n.d.	[39]
PpcABCDE^ox^	CbcA^red^	+	n.d.	[39]
PpcA^ox^	CbcS^red^	+	n.d.	[77]
PpcA^ox^	ImcH^red^	+	Heme I and IV (PpcA)	[81]
PpcABCDE^ox^	MacA^red^	+	Heme IV (PpcA,B,D and E) Heme I (PpcC)	[92]
PpcABCDE^ox^	OmcS^red^	+	Hemes III and IV (PpcA) Heme I (PpcB) Hemes I and IV (PpcC,D and E)	[75]

^ox^ and ^red^ indicate the initial oxidation state of each cytochrome used in the ET assays. (+), ET detected; (−), no ET detected; n.d., not determined.

## Perspectives

- The unique respiratory capabilities of electrogenic bacteria have paved the way for a wide range of important environmental processes and biotechnological applications for bioenergy, bioremediation, and bioelectronics, whose further optimization relies on a detailed comprehension of the underlying electron transfer mechanisms.
- The current model for ET pathways in the electrogenic bacterium *Geobacter sulfurreducens* involves the formation of weak-affinity and transient redox complexes between distinct *c*-type cytochromes across the cellular compartments, enabling a smooth control of the directionality of the ET and flexible adaptation to environmental redox changes.
- The clarification of the entire set of redox partners involved in the ET networks together with the elucidation of the electron conductive mechanisms through the microbial nanowires will enable the rational design of more efficient biotechnological applications.

## Open Access

Open access for this article is provided via our Subscribe to Open programme, made possible through the support of our subscribing institutions. Learn more here: https://portlandpress.com/pages/open_access_options_and_prices#conditional.

## Abbreviations

CSP: chemical shift perturbation
EET: extracellular electron transfer
ET: electron transfer
*Gs*: *Geobacter sulfurreducens*
IM: inner membrane
MQ: menaquinone
OM: outer membrane
